# Biodiversity is decimated by the cascading effects of the amphibian-killing chytrid fungus

**DOI:** 10.1371/journal.ppat.1010624

**Published:** 2022-07-21

**Authors:** Elise F. Zipkin, Graziella V. DiRenzo

**Affiliations:** 1 Department of Integrative Biology; Ecology, Evolution, and Behavior Program, Michigan State University, East Lansing, Michigan, United States of America; 2 U.S. Geological Survey, Massachusetts Cooperative Fish and Wildlife Research Unit, University of Massachusetts, Amherst, Massachusetts, United States of America; Geisel School of Medicine at Dartmouth, UNITED STATES

## What is the *Batrachochytrium dendrobatidis* (Bd) fungus?

*Batrachochytrium dendrobatidis* (Bd) is a water-borne chytrid fungus that infects keratinized tissues of its amphibian hosts [1]. Specifically, Bd infects the skin of subadult and adult amphibians and the mouthparts of larval amphibians, and often causes host mortality via its effects on osmotic balance [2]. Bd has a two-part lifecycle. First, the aquatic, flagellated zoospore is the infective stage, which encounters the amphibian’s keratinized skin and encysts within epithelial cells. Once embedded, the zoospore develops into the reproductive stage, known as the zoosporangium, which produces more zoospores and releases them onto the skin of the amphibian and into the environment within days to weeks [3–5].

Bd transmission occurs through direct contact with infected individuals [6] and indirect contact with infected materials [7]. To date, there are no confirmed environmental reservoirs of Bd with documented signs of saprophytic growth outside the amphibian host; however, Bd DNA has been detected on a number of non-amphibian species and materials (e.g., [8]).

An interesting aspect of Bd biology is that it contains characteristics of both a microparasite and macroparasite [9]. Although Bd is small-like microparasites, host infection intensity (i.e., number of zoospores) is predictive of disease outcome and infectivity, which is common of macroparasites. However, unlike many microparasites, Bd lacks an efficient way to transmit between cells within an infected host (although transmission between cells has been documented [10,11]). Increasing infection intensity generally depends on external reinfection, which is a general characteristic of macroparasites. In the case of Bd, zoospores are released from within the skin and onto the skin surface to reinfect a host.

## Which species are directly impacted by Bd-related chytridiomycosis?

**Fig 1:**
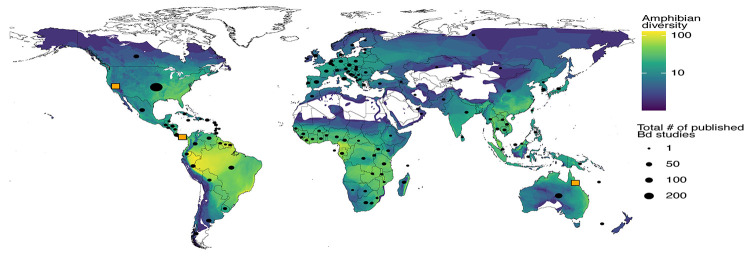
Global map of amphibian diversity (i.e., number of species), the number of Bd studies published per country, and locations with species data before and after a Bd-related chytridiomycosis epizootic. Orange squares show the worldwide locations of studies that contain data on amphibians as well as other taxonomic groups before and after Bd invasion. Amphibian biodiversity data were obtained from [49] and the number of published Bd studies per country was tabulated from [15].

Mortality by Bd-related chytridiomycosis has only been directly observed in amphibians [12]. There are more than 8,400 described amphibian species [13], most of which have never been sampled for Bd. Of the 2,525 amphibian species sampled for Bd, 1,375 species (55%) contain at least one individual that tested positive [14]. A recent global assessment documented that Bd has influenced the decline of at least 500 amphibian species, including the extinction of 90 species [14]. Bd has been detected in 93 of the 134 countries (69%) where sampling has occurred [15]. Although these values are high, they are likely underestimated because of unequal Bd sampling efforts across the globe and species (Fig 1).

### Among species variation

Since the discovery of Bd in the late 1990s, scientists quickly documented variation in the susceptibility of amphibian species to Bd infection and mortality [16–18]. Upon Bd exposure, some amphibian species become infected quickly and are extirpated immediately, other species decline more gradually, while a smaller number persist post pathogen invasion [19,20]. Variations in amphibian species susceptibility to and mortality from Bd infection have been linked to a number of abiotic (e.g., temperature, habitat type, salinity) and biotic (e.g., amphibian community composition, species life history) factors (reviewed by [21]). For example, Bd does not equally affect amphibians across ecological guilds [22] or geographic regions [23], and Bd disproportionately affects low-occurrence, endemic species [24] and species with narrow elevational ranges [14]. Although it has been difficult to parse “the signal from the noise” as there is tremendous variation in species-level susceptibility to Bd, some characteristics that have been associated with severe population-level declines are large body size, consistent occurrence in wet regions, and strong associations with perennial aquatic habitats [14,17].

### Within species variation

Even within amphibian species, there are large variations in susceptibility among individuals and populations. Differences in genotype (e.g., [25]), microbiome (e.g., [26]), and host immune responses (e.g., [27–29]) can lead to variations in Bd susceptibility. Alternatively, the variation in susceptibility between populations within species may be largely driven by variation in Bd virulence among strains [30]. Like most host–pathogen systems, the patterns of Bd infection and susceptibility within and among species are shaped by the interactions among the pathogen, host, and environmental variables, although the specific details of these interactions are complex and not well understood.

## What are the indirect consequences of Bd-related chytridiomycosis epizootics?

Severe declines in the abundance and species richness of amphibians from Bd can have substantial cascading effects on other aspects of an ecosystem’s structure and function, including the depletion or loss of other taxonomic groups. Yet, there are many challenges to documenting biodiversity changes following a Bd-related chytridiomycosis epizootic. First, it is difficult to know where and when a chytridiomycosis epizootic will take place and, as a result, baseline biodiversity data (prior to an epizootic) is typically unavailable (Fig 1). Ideal baseline data include long-term information on the status (and trends) of amphibian species as well as other taxonomic groups, which typically requires the coordination of large teams of biologists. To date, only three locations worldwide have such data available (to varying degrees): tropical rainforests near El Copé, Panama; montane rainforests in Queensland, Australia; and the Sierra Nevada mountains in central California, United States (Fig 1). Thus, our knowledge on the consequences of widespread amphibian losses from Bd-related chytridiomycosis is fairly limited in scope, and as a result, the scientific literature likely underestimates the true extent of the problem within and across ecosystems.

**Fig 2:**
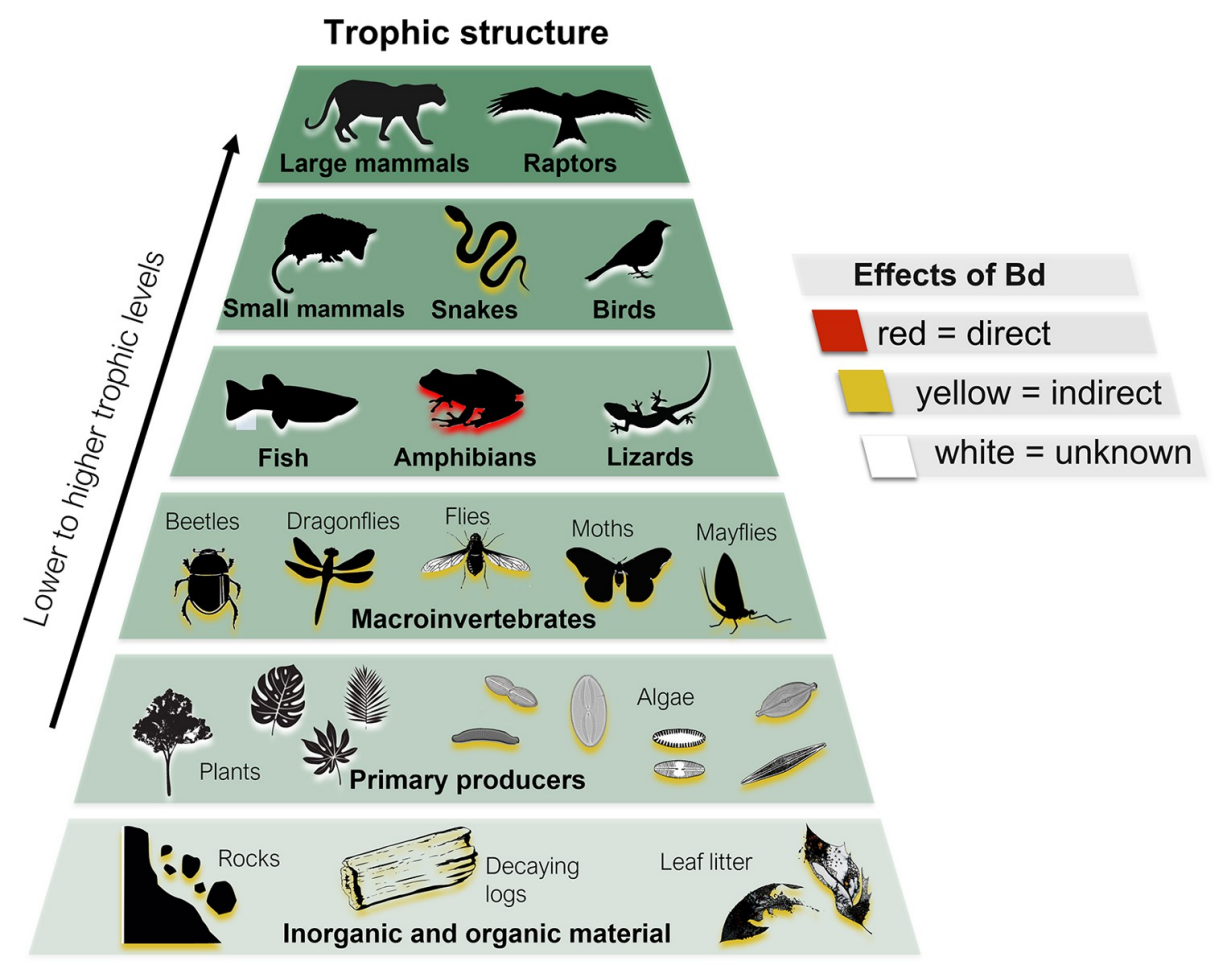
Trophic diagram showing the cascading effects of the Bd invasion in El Copé, Panama. Following the arrival of Bd, amphibians experienced mass mortality from the direct effects of the disease (shadowed in red), which led to significant changes across the local ecosystem through cascading processes. Studies have documented both indirect bottom-up and top-down effects of Bd, including changes in the richness and composition of snakes as well as in the structure and functional diversity of macroinvertebrates, primary producers, and inorganic and organic material (shadowed in yellow). Additional taxa in the system (shadowed in white) could have also been impacted by the Bd invasion, but to date, there are no published studies on these taxonomic groups. Species groups shown at the various tropic levels are representative and not exhaustive.

Because many amphibians have both aquatic (e.g., tadpole) and terrestrial (e.g., adult) life stages, their losses have the potential for wide ranging top-down and bottom-up impacts within ecosystems (Fig 2; [31]). Unsurprisingly, the consequences of amphibian declines appear to be most severe in areas where the abundance and species richness of amphibians are high (e.g., the tropics). For example, in streams where tadpoles are dominant grazers, researchers have documented top-down declines in whole stream respiration from reduced nitrogen uptake and deposited organic sediment as a result of increased algae and detritus biomass following Bd-related amphibian losses [32,33]. Tadpole abundance can also influence leaf decomposition rates through their effects on microbial communities [34], as well as the abundance and diversity of macroinvertebrates [35]. When tadpoles are lost from these aquatic ecosystems, food web structures can rearrange, showing high adaptive capacities [36,37]. However, deficiencies are not always compensated by other taxa, even after long time frames, and significant changes to ecosystem structures and functions can persist [35].

The loss of amphibians (both tadpoles and adults) can also have significant bottom-up effects on other taxonomic groups that prey on amphibians [38,39]. In many places where amphibians occur, adult amphibians are preyed upon by carnivorous animals, including snakes and other reptiles, mammals, and raptors. For example, in California, garter snakes declined following amphibian mass mortality from a Bd-related chytridiomycosis epizootic [38], and, in Panama, where snake richness and diversity are quite high, the loss of amphibians led to a >20% decline in the estimated richness of local snake species [40]. Surprisingly, there was only anecdotal evidence that the snake species most severely impacted by amphibian losses had diets that heavily relied on amphibian prey [41]. Some snake species that were thought to be generalists also decreased in occurrence, suggesting that there may be other, indirect effects of Bd epizootics that are not easily measured. Because many tropical species are rare, and thus difficult to sample, the true rates and mechanisms of biodiversity loss caused by Bd-related chytridiomycosis epizootics are impossible to thoroughly document (Fig 2).

## How is the Bd-related panzootic contributing to biotic homogenization?

Biotic homogenization is the increase in taxonomic similarity among distinct, geographically separated regions and the loss of biological differences in any organizational level (e.g., population, community) in terms of functional, taxonomic, or genetic features [42]. Biotic homogenization can decrease biodiversity, increase incidence and distribution of infectious disease, and reduce resiliency to ecosystem-level disturbances [43]. Because the loss of amphibians from Bd is not random across and within species, we tend to find biotic homogenization occurring in amphibian communities following a Bd invasion, resulting in reduced taxonomic diversity, in which common species persist [37,44]. Interestingly, the indirect impacts of a Bd-related chytridiomycosis epizootic have a similar nonrandom effect on other taxonomic groups impacted by the loss of amphibians. For example, within macroinvertebrate communities, filter-feeding, grazing, and shredding species decreased immediately following a Bd epizootic, while collector–gatherer species increased during the same time period [35]. In this same community, a few snake species maintained their pre-Bd occurrence rates or even increased despite the fact that most snake species occurrence rates decreased following the loss of amphibians [40]. Certain species traits or adaptive capacities may be more universally resistant to ecological disturbances than others, such that the cascading consequences of a Bd-related chytridiomycosis epizootic can lead to more similar, regional-level species pools and attributes of biodiversity across ecosystems and regions. Perhaps the biggest unanswered questions related to the amphibian-killing chytrid fungus lie in identifying the mitigating factors that lead to the resiliency of some individuals and species and the demise of others across the ecosystem. Determining the extent to which these factors are genetic versus environmental and whether there is any universality in the cascading effects of the Bd-amphibian system across geographic locations can aid in the prevention and mitigation of downstream biodiversity loss.

## How can we mitigate biodiversity loss from Bd-related chytridiomycosis epizootics?

The extirpation of a single species or a change in community composition can have cascading impacts throughout an ecosystem [45,46]. Detecting and documenting the consequences of biodiversity changes are logistically challenging because it is difficult to predict the location and timing of mass mortality events caused by epizootics, species invasions, and climate change. Making matters more difficult, many species worldwide are data deficient, providing an insufficient reference by which to evaluate ecological change. In an effort to mitigate future biodiversity loss, conservation biologists and managers can take a proactive rather than reactive approach [47]. For example, predicting changes in local-scale biodiversity patterns under global change scenarios (e.g., [48]) can help prioritize where and when to collect baseline data before losses occur. Thorough assessments of the statuses and trends of known species and whole communities are clear priorities, especially in regions that are understudied and/or have high levels of biodiversity. Research on the cascading impacts of the Bd-amphibian system has helped demonstrate that the consequences of disease invasions can extend well beyond those species that are directly infected by the pathogen, leading to the decline of species that are considered only tangentially connected to the system (e.g., [40]). In the case of fungal pathogens, the salamander-killing fungus *Batrachochytrium salamandrivorans* (Bsal) poses a threat similar to that of its sister lineage Bd, which wreaked havoc several decades prior. Thus, there is a unique opportunity to study these taxonomic groups, understand their roles within ecosystems, and develop strategies to limit the spread of Bsal. Together, these efforts are critical to mitigating biodiversity loss in the near term and preventing further losses in the future.
